# Likelihood and Predictors of ST-Elevation in Patients Hospitalized for Myocardial Infarction

**DOI:** 10.1371/journal.pone.0108440

**Published:** 2014-09-25

**Authors:** Ville Kytö, Jussi Sipilä, Päivi Rautava

**Affiliations:** 1 Heart Center, Division of Clinical Neurosciences, Neurology and Clinical Research Center, Turku University Hospital, Turku, Finland; 2 PET Center, Medicine, Neurology and Public Health, University of Turku, Turku, Finland; Morehouse School of Medicine, United States of America

## Abstract

**Importance:**

Emergency treatment options in myocardial infarction are guided by presence or absence of ST-elevations in electrocardiography. Occurrence and factors associated with ST-presentation in different population groups are however inadequately known.

**Objective:**

To determine likelihood and patient features associated with ST-elevations in myocardial infarction.

**Design:**

Nationwide registry study including 22 hospitals with angiolaboratory during an eight year period in Finland.

**Setting:**

Hospitalized care.

**Participants:**

68,162 consecutive patients aged ≥30 with myocardial infarction.

**Measures:**

Likelihood and patient features associated with presence of ST-elevations.

**Results:**

Myocardial infarction presented with ST-elevation in 37.5% (CI 37.0–37.9%) and without in 62.5% (CI 61.9–63.1%) of patients, p<0.0001. Majority of patients aged 30–59 years with myocardial infarction had ST-elevation, but among octogenarians ST-elevations were present in only 24.7%. Presence of ST-elevations decreased with age by estimated 15.6% (CI 15.0–16.2%) per 10 year increase (p<0.0001). Men aged 40–79 years had significantly higher rate for ST-elevation myocardial infarction compared to women. Sex-based difference in presentation of myocardial infarction declined with increasing age. Overall, men had a 13% (CI 11–15%, p<0.0001) higher relative risk for ST-elevations compared to women when adjusted for age and co-morbidities. Diabetes, atrial fibrillation, peripheral or cerebral artery disease, chronic pulmonary disease, malignancy, and renal insufficiency were associated with absence of ST-elevations in myocardial infarction in multivariate analysis.

**Conclusions and Relevance:**

Myocardial infarction presents with ST-elevations more commonly in men. Presence of ST-elevations decreases with increasing age. Diabetes, atrial fibrillation, peripheral or cerebral artery disease, chronic pulmonary disease, malignancy, and renal insufficiency are associated with absence of ST-elevations in myocardial infarction. These findings may help to predict likelihood of ST-elevations in a patient with myocardial infarction.

## Introduction

Emergency treatment strategies of patients with myocardial infarction (MI) are based on ST-segment presentation in electrocardiography. In ST-elevation myocardial infarction (STEMI), an acute total coronary occlusion is present and immediate reperfusion therapy, preferably by primary percutaneous coronary intervention, is required [1], [2]. When ST-elevations are not present, but circulating troponin levels are elevated, patient has a non-ST-elevation myocardial infarction (NSTEMI) requiring intensive medical therapy and invasive assessment of coronary arteries should be conducted during the next 24 hours [3], [4]. Although the majority of all MI patients are known to have NSTEMI [5], factors associated with ST-presentations are less well known. We studied the likelihood of ST-segment elevation in myocardial infarction and patient features predicting it using a large multihospital registry.

## Methods

### Study Patients and Data Collection

We studied 68,162 consecutive patients aged ≥30 years admitted to hospital with myocardial infarction as primary discharge diagnosis (ICD-10 code I21) in 22 hospitals during a period of 8 years. Infarction was classified as STEMI or NSTEMI based on ICD-10 coding (I21.0x–I21.3x vs. I21.4x–I21.9x, respectively). Data was retrospectively collected from the Finnish Hospital Discharge Register, a nationwide database maintained by the Finnish National Institute for Health and Welfare containing hospital discharge diagnosis codes (ICD-10) of all medical admissions in Finland. Hospital transfers (10.8% of admissions) during the same treatment period were combined as one. All 22 hospitals in Finland that treat emergency patients and have a coronary catheterization laboratory were included. Admissions that begun between January 1^st^ 2001– December 31^st^ 2008 were included. The study was approved by the National Institute for Health and Welfare (permission nro THL/1576/5.05.00/2010). Patient data was received anonymized and de-identified and informed consents were thus not obtained.

### Statistical Analysis

Scale variables are presented as mean±SD or median with interquartile range (IQR) as appropriate. Categorical variables are presented as percentages or relative risks (RR) with 95% confidence interval (95% CI) as appropriate. Differences in continuous variables were analyzed with student t-test. Overall distribution of myocardial infarction by presenting ST-change was tested with Chi-square test. Factors associated with the type of myocardial infarction were studied by using a log-binomial regression model. Patient characteristics associated with type of infarction at p<0.05 in univariate analysis (stratified for study year) were included in the final regression model. Two-sided p-values <.05 were considered statistically significant. The SAS system (v.9.3, SAS Institute Inc, Cary, NC, USA) was used for statistical analyses.

## Results

### Incidence of ST-Elevations in Myocardial Infarction

**Figure 1:**
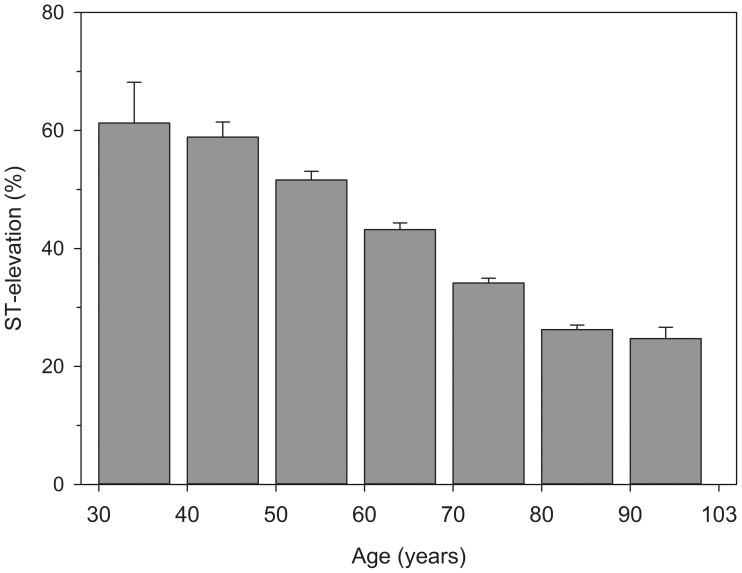
Proportion of patients with ST-elevation myocardial infarction by age. Error bars represent upper limits of 95% confidence interval.

**Figure 2:**
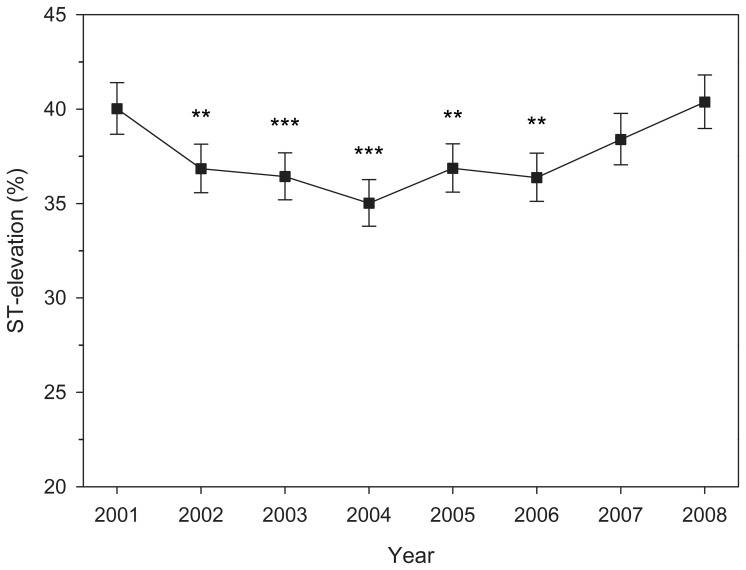
Annual variation of the presence of ST-elevation in patients with myocardial infarction. Error bars represent 95% confidence interval. ** p<0.005, *** p<0.0005 (vs. year 2001). Please note the origin of y-axis.

Myocardial infarction presented with ST-elevation in 25,538 patients (37.5%; CI 37.0–37.9%) and without ST-elevation in 42,624 patients (62.5%; CI 61.9–63.1%), p<0.0001. Mean age of all patients was 71.2±12.6 years. STEMI patients were significantly younger than patients with NSTEMI (67.7±13.0 vs. 73.3±11.8 years, p<0.0001). Presence of ST-elevations in MI decreased with age (Figure 1). Majority of patients aged 30–59 years of age had ST-elevation MI, but of octogenarians only 24.7% (CI 22.9–26.6%) had STEMI. Although the proportion of STEMI did not differ between beginning and end of study period, there was significant annual variation during the study period (Figure 2). Proportion of MI with ST-elevation decreased by estimated 3.3% (CI 2.2–4.4%) annually during 2001–2004 (p<0.0001) followed by an estimated increase of 3.2% (CI 2.3–4.0%, p<0.0001) per year from 2004 onwards.

### Patient Characteristics Associated with ST-Elevation

**Figure 3:**
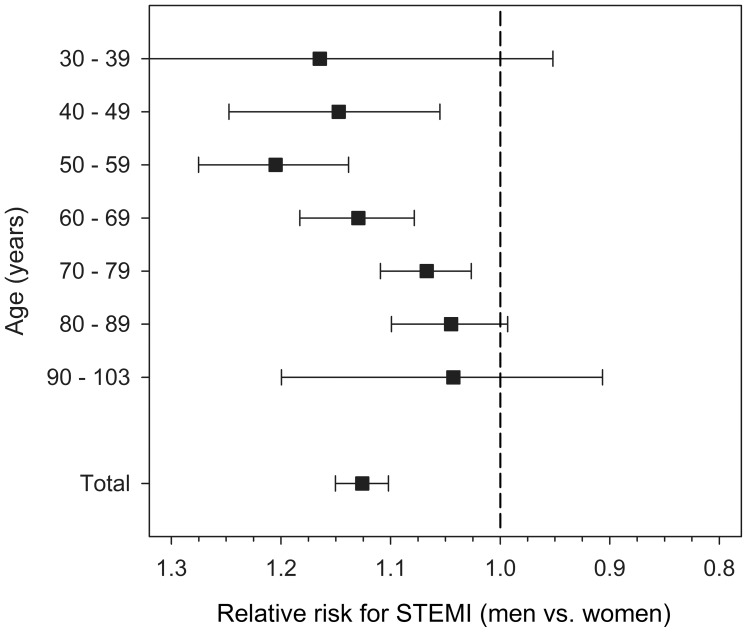
Sex-related likelihood of ST-elevations (STEMI) in myocardial infarction by age. Relative risk is calculated as men vs. women and adjusted for significant baseline characteristics. Error bars represent 95% confidence interval.

Majority of both STEMI and NSTEMI patients were male (Table 1). Men had a 32% (CI 30–35%, p<0.0001) higher unadjusted rate for ST-elevations compared to women. Sex-based likelihood for ST-elevation varied however significantly with age, as men aged 40–79 had significantly higher rate than women, and sex-based likelihood decreased with increasing age (Figure 3). After adjustment for age and other characteristics, the overall rate of ST-elevations was 13% (CI 11–15%, p<0.0001) higher in men. The adjusted likelihood of ST-elevation decreased by 15.6% (CI 15.0–16.2%) per 10 increase in age (p<0.0001). Diabetes, atrial fibrillation, peripheral or cerebral artery disease, chronic pulmonary disease, malignancy, and renal insufficiency were all associated with absence of ST-elevation in MI in both univariate (Table 1) and multivariate analyses (Table 2).

**Table 1:** Characteristics of myocardial infarction patients with (STEMI) and without ST-elevation (NSTEMI).

	Prevalence % (95% CI)		Association with ST-elevation *	
	STEMI	NSTEMI	RR	p
Male sex	66.3 (65.3–67.4)	55.8 (55.1–56.5)	1.32 (1.30–1.35)	<0.0001
Hypertension	13.5 (13.0–13.9)	13.5 (13.2–13.9)	1.00 (0.97–1.03)	0.9508
Diabetes	7.1 (6.8–7.4)	8.4 (8.1–8.7)	0.89 (0.85–0.92)	<0.0001
Atrial fibrillation	4.4 (4.1–4.7)	8.2 (8.0–8.5)	0.63 (0.60–0.67)	<0.0001
Peripheral or cerebral artery disease	2.0 (1.8–2.2)	2.7 (2.5–2.8)	0.83 (0.77–0.89)	<0.0001
Chronic pulmonary disease	1.8(1.6–2.0)	2.5 (2.4–2.7)	0.80 (0.74–0.86)	<0.0001
Malignancy	0.9 (0.8–1.0)	1.2 (1.1–1.3)	0.83 (0.74–0.93)	0.0008
Renal insufficiency	0.6 (0.5–0.7)	1.0 (0.9–1.1)	0.74 (0.64–0.84)	<0.0001
Rheumatoid arthritis	0.5 (0.4–0.6)	0.5 (0.5–0.6)	0.94 (0.81–1.08)	0.3696

*Univariate analysis, stratified for study year.

Association of characteristics with ST-elevation myocardial infarction in univariate regression analysis (stratified for year). RR = relative risk. 95% CI = 95% confidence interval.

**Table 2:** Association of patient features with ST-elevation in myocardial infarction in multivariate analysis.

	RR (95% CI)	p
Male sex	1.13 (1.10–1.15)	<0.0001
Age (10 year groups)	0.84 (0.84–0.85)	<0.0001
Diabetes	0.89 (0.86–0.93)	<0.0001
Atrial fibrillation	0.73 (0.69–0.77)	<0.0001
Peripheral or cerebral artery disease	0.90 (0.84–0.97)	0.0054
Chronic pulmonary disease	0.84 (0.78–0.91)	<0.0001
Malignancy	0.88 (0.79–0.98)	0.022
Renal insufficiency	0.84 (0.74–0.96)	0.0089

RR = relative risk. 95% CI = 95% confidence interval.

## Discussion

This multi-hospital study of all-comer MI patients describes likelihood and patient characteristics associated with ST-elevations in myocardial infarction. In agreement with previous studies [5] we found the majority of all MIs to be NSTEMI. However, MI presented with ST-elevations more commonly than without in patients less than 59 years of age, and the proportion of STEMI declined with increasing age. This finding may reflect the fact that prevalence of chronic coronary artery disease (CAD) increases with aging [5], [6] and the occlusion of a small, already stenotic small coronary side branch will not result in ST-elevation in electrocardiography. Furthermore, presence of already developed coronary collaterals in patients with stable coronary disease may prevent total ischemia and ST-elevations in vascular territory of totally occluded coronary [7], [8]. Age-related changes in platelet structure and function [9], [10] may also contribute for decreasing proportion of STEMI with age, but significance and pathophysiology of age-related platelet function and thrombogenesis are yet unclear.

Previous studies have found males to be overrepresented by 5–9% among STEMI patients compared to NSTEMI [11]–[17]. Sex-based likelihood for ST-elevation is however rarely presented in literature. A study using Euro-Heart Survey 2004 data found women under 65-years of age to have an odds ratio of 0.62 for ST-elevation compared to men while sex did not affect the risk in older patients, but results were not adjusted for co-founders [18]. We found men to have a relative risk of 1.13 for ST-elevations compared to women after adjustment for age and co-morbidities. Reasons for this gender-bias are unknown, but may include differences in platelet function [19], coagulation-fibrinolytic pathway [20], inflammatory response [21], coronary plaque composition [22], matrix remodeling [23], endothelial function [24], and angiogenesis [25] in addition to lifestyle and risk factors differences [26]. Estrogen is found to play a pivotal role in sex-differences in coronary artery disease [19], [23]–[25], [27]. In agreement, we found the sex-based likelihood for ST-elevation in myocardial infarction to decrease with increasing age.

We found the known markers for the likelihood of CAD, such as atherosclerotic disease of cerebral or peripheral arteries, diabetes, chronic pulmonary disease, and renal insufficiency [5] to be associated with absence of ST-elevations. This most likely reflects the protective role of coronary collaterals [8] from ST-elevation in addition to an increased likelihood for small side branch occlusion. In addition to a higher likelihood for development of chronic CAD [5], diabetes has multiple effects on risk of myocardial infarction including alterations in artery wall and plaque remodeling [28] and thrombogenesis [29]. Atrial fibrillation was also associated with MI without ST-elevations in our study. This may be due to ongoing oral anticoagulation therapy for stroke prevention [30] that also prevents formation of totally occlusive coronary thrombus. Our finding of association between malignancy and absence of ST-elevations is in agreement of a previous study of MI in cancer patients [31], and is likely to be due to cancer related abnormalities in coagulation and thrombosis [32], and the effects of anticancer therapies [33].

Previous studies originating from US [11], Ireland [14], and Italy [34] have reported constantly declining proportions of STEMI among MI patients in recent years. We found the proportion of STEMI to slightly decline during 2001–2004, but contradictory to previous findings, proportion of STEMI steadily increased from 2004 onwards. Reasons for this discrepancy require further study, but the end in the decline of blood pressures in addition to increase in obesity [26] and binge drinking [35] during recent years in Finland are potential contributors.

The current study has limitations associated with use of retrospective hospital registry data. Thus, diagnoses were made by treating physicians, which may have affected the included patient population. Hospital discharge data has, however, proved to be a valuable source of information on cardiovascular disease [36]. The nationwide hospital discharge registry utilized here is government maintained, automatic, and mandatory thus capturing information of all hospital admissions and resulting in accurate information on cardiovascular disease [37], [38]. In addition to a small number of inevitable coding inaccuracies [38], diagnostic inaccuracies may be a potential source of error. As treatment choices in MI rely heavily on distinction between STEMI and NSTEMI, the correct interpretation of electrocardiography and resulting diagnostic classification are however major priorities among physicians treating patients with an acute coronary syndrome. In order to minimize misdiagnosis of MI in chest pain patients with non-coronary causes such as extra-cordial pain, myocarditis, aortic dissection, or pulmonary embolism [39], [40] we included only patients treated in hospitals with coronary angiolaboratory. Therefore, patients who did not reach hospital due to e.g. sudden cardiac death associated with myocardial infarction were not included.

In conclusion, myocardial infarction presents with ST-elevations more commonly in men and the presence of ST-elevations decreases with increasing age. Diabetes, atrial fibrillation, peripheral or cerebral artery disease, chronic pulmonary disease, malignancy, and renal insufficiency are associated with absence of ST-elevations in myocardial infarction. These findings may help to predict likelihood of ST-elevations in a patient with myocardial infarction.
